# Causal-ARG: a causality-guided framework for annotating properties of antibiotic resistance genes

**DOI:** 10.1093/bioinformatics/btae180

**Published:** 2024-04-03

**Authors:** Weizhong Zhao, Junze Wu, Xingpeng Jiang, Tingting He, Xiaohua Hu

**Affiliations:** Hubei Provincial Key Laboratory of Artificial Intelligence and Smart Learning, Central China Normal University, Wuhan, Hubei 430079, P.R. China; School of Computer, Central China Normal University, Wuhan, Hubei 430079, P.R. China; National Language Resources Monitoring & Research Center for Network Media, Central China Normal University, Wuhan, Hubei 430079, P.R. China; Hubei Provincial Key Laboratory of Artificial Intelligence and Smart Learning, Central China Normal University, Wuhan, Hubei 430079, P.R. China; Hubei Provincial Key Laboratory of Artificial Intelligence and Smart Learning, Central China Normal University, Wuhan, Hubei 430079, P.R. China; Hubei Provincial Key Laboratory of Artificial Intelligence and Smart Learning, Central China Normal University, Wuhan, Hubei 430079, P.R. China; College of Computing & Informatics, Drexel University, Philadelphia, PA 19104, United States

## Abstract

**Motivation:**

The crisis of antibiotic resistance, which causes antibiotics used to treat bacterial infections to become less effective, has emerged as one of the foremost challenges to public health. Identifying the properties of antibiotic resistance genes (ARGs) is an essential way to mitigate this issue. Although numerous methods have been proposed for this task, most of these approaches concentrate solely on predicting antibiotic class, disregarding other important properties of ARGs. In addition, existing methods for simultaneously predicting multiple properties of ARGs fail to account for the causal relationships among these properties, limiting the predictive performance.

**Results:**

In this study, we propose a causality-guided framework for annotating properties of ARGs, in which causal inference is utilized for representation learning. More specifically, the hidden biological patterns determining the properties of ARGs are described by a Gaussian Mixture Model, and procedure of causal representation learning is used to derive the hidden features. In addition, a causal graph among different properties is constructed to capture the causal relationships among properties of ARGs, which is integrated into the task of annotating properties of ARGs. The experimental results on a real-world dataset demonstrate the effectiveness of the proposed framework on the task of annotating properties of ARGs.

**Availability and implementation:**

The data and source codes are available in GitHub at https://github.com/David-WZhao/CausalARG.

## 1 Introduction

Since the discovery of penicillin, antibiotics have consistently played a powerful role in the human effort to combat bacterial infections. (Stokes *et al.* 2020). However, due to the misuse and overuse of antibiotics, the bacterial antimicrobial resistance (AMR)—which refers to the ability of bacteria to resist the effects of antimicrobial drugs, such as antibiotics—has emerged as one of the leading public health threats of the 21st century (Murray *et al.* 2022). It is widely known that bacteria acquire antibiotic resistance through antibiotic resistance genes (ARGs) or the proteins they encode within bacteria (Amarasiri *et al.* 2020). Therefore, accurately annotating ARGs can help us better understand antibiotic resistance and explore new therapeutic strategies (Florensa *et al.* 2022).

Monitoring and comprehending the mechanisms and the spread of antimicrobial resistance are crucial for both personal healthcare and global infection control strategies (Boolchandani *et al.* 2019). Among the various properties of ARGs, the most meaningful for clinical treatment and biological research are antibiotic class, resistance mechanism, and transferability. For each ARG, understanding the specific antibiotic class can help us treat antibiotic-resistant bacterial infections more effectively (Jian *et al.* 2021). Since different ARGs might possess different resistance mechanisms, exploring antibiotic resistance mechanisms provides fundamental insights into underlying patterns controlling the development of ARGs (Darby *et al.* 2023). In addition, resistance to antibiotics can occur either by mutations or by acquisition of resistance conferring genes via horizontal gene transfer (HGT), of which the latter is considered to be the most important factor in the current pandemic of AMR (Von Wintersdorff *et al.* 2016). For example, bacteria containing the antibiotic resistance gene CTX-M (Cantón and Coque 2006) exhibits high resistance to β-lactam antibiotics, and understanding this characteristic helps avoid ineffective treatments. Moreover, CTX-M encodes β-lactamase which can inactivate β-lactam, and understanding this mechanism helps reveal how the CTX-M gene confers antibiotic resistance to bacteria, providing a foundation for developing new antibiotics or improving treatment strategies. In addition CTX-M is typically located on mobile plasmids, facilitating the HGT between bacteria, and understanding this property is crucial for preventing the spread of antibiotic resistance caused by CTX-M. Therefore, the primary objective of annotating an ARG is to describe its antibiotic class, resistance mechanism, and transferability. These aspects are also the central focus of our research in this study.

For the task of annotating properties of ARGs, a typical input is the sequence of potential ARG-encoded protein, and the output is the corresponding predicted properties. Given each sample *X*, the corresponding ground-truth label *Y* is a tuple $\left(Y_{a},Y_{m},Y_{t}\right)$ indicating the antibiotic class, resistance mechanism and transferability, respectively. More specifically, $Y_{a}\in \{1,\ldots ,C^{A}\}$ and $Y_{m}\in \{1,\ldots ,C^{M}\}$, where $C^{A}$ and $C^{M}$ represent the total number of antibiotic classes and resistance mechanisms, respectively. In addition, $Y_{t}$ can only be 0 or 1, with ‘1’ indicating that the antibiotic resistance is acquired through horizontal gene transfer and ‘0’ indicating that the antibiotic resistance is intrinsic in the bacteria. Given a training set *S* of *N* samples (i.e. $S=\{(X_{1},Y_{1}),\ldots ,(X_{n},Y_{n}),\ldots ,(X_{N},Y_{N})\}$ where $X_{n}$ denotes the *n*th sample and $Y_{n}=(Y_{n,a},Y_{n,m},Y_{n,t})$ denotes the corresponding ground-truth label), a predictor or annotator will be trained, by which the properties of a new potential ARG can be predicted accordingly based on the protein sequence of the input gene.

Metagenomic (Simon and Daniel 2011) and high-throughput sequencing technology (Dominguez-Bello *et al.* 2011) induced the advent of several tools for analysis of ARGs including BestHit (Alcock *et al.* 2020). Since it relies on alignment with existing databases, the BestHit method usually requires extended timeframes and substantial costs. In addition, the BestHit method has the following limitations: (i) its performance relies significantly on the quality and completeness of the database. If certain types of antibiotic resistance genes are missing in the database, the BestHit method may fail to accurately predict these genes. (ii) The BestHit method typically selects only the most similar sequence as the best match, potentially overlooking other sequences with relatively lower similarity. This may result in a higher false-negative rate. With the rapid development of deep learning technologies in recent years, deep learning has also found widespread applications in the field of bioinformatics. Researchers have proposed several deep learning-based models for annotating ARG properties. Arango-Argoty *et al.* (2018) developed DeepARG which annotates ARGs using similarity features by comparing the query sequence to the existing ARG databases. Li *et al.* (2021) proposed a hierarchical multi-task deep learning model for annotating antibiotic resistance genes (HMD-ARG), in which predicting antibiotic classes, resistance mechanisms, and transferability are regarded as three subtasks for potential antibiotic resistance genes. In DeepARG, a simple multi-layer fully connected network is utilized to acquire the representations of ARGs, while in HMD-ARG, Convolutional Neural Networks (CNNs) are utilized to acquire the representation of ARGs.

However, existing deep learning-based methods for annotating ARG properties have the following two main drawbacks:

1. Deep learning models typically use an end-to-end manner to learn latent representations from input sequences, and establish correlations between these representations and various properties of ARGs. However, due to the complex biological patterns behind antibiotic resistance, the representations obtained by these methods may fail to fully describe the characteristics of ARGs, leading to the suboptimal prediction performance. For instance, for ‘L-form’ bacteria (Claessen and Errington 2019), the cell wall dissolves under high osmotic pressure conditions, which renders antibiotics targeting the cell wall ineffective and leads to bacterial resistance. The occurrence of such antibiotic resistance cannot be captured through analyzing only the sequence information of ARGs.
2. Most of existing methods can only annotate one specific property (such as antibiotic class) while annotating resistance mechanisms and transferability is equally crucial. Although certain method like HMD-ARG can predict the three properties simultaneously, it simply treats the prediction of the three properties as three separate subtasks, which may lead to a problem called the “seesaw” phenomenon (Zhang *et al.* 2022), i.e. it performs well on certain subtasks while deriving poor performance on others. Furthermore, existing methods ignore the possible causal relationships between different properties of ARGs, restricting further improvement in their performance.

Based on the above observations, we propose a novel ARG properties annotation framework called Causal-ARG, in which causal inference knowledge is used to address the above limitations of existing methods. As far as we know, Causal-ARG is the first attempt to incorporate causal knowledge into the annotation of ARG properties. More specifically, we introduce a causal representation learning module to learn the latent representation of ARGs, which is able to capture the comprehensive semantics contained in the complex biological patterns of bacterial antibiotic resistance. In addition, we use a causal relationship mining tool to construct a causal graph among the three properties of AGRs. When predicting the properties of ARGs, we integrate the derived causal graph to improve the prediction performance. Experimental results on a real-world data demonstrate that the proposed method outperforms the state-of-the-art methods on annotating ARGs’ properties, and the ablation experiments validate the effectiveness of the main components in our model as well. Moreover, the results of case study verify the effectiveness and usability of the proposed method.

## 2 Materials and methods

**Figure 1.**
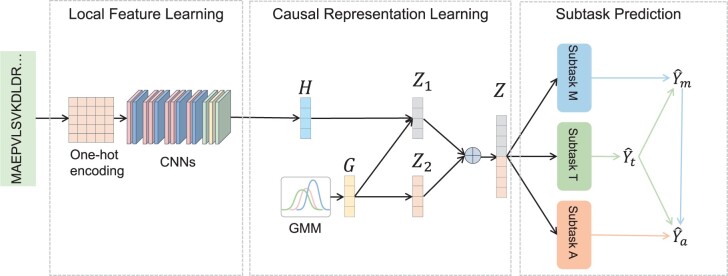
Overview of Causal-ARG. Local Feature Learning: multiple layers of CNNs are utilized to learn local representation *H* from the input sequence information. Causal Representation Learning: a causality-guided representation learning module is to integrate the complex biological patterns of bacteria antibiotic resistance. Subtask Prediction: a causal graph among different properties is constructed to improve prediction performance.

The schematic representation of the proposed framework is shown in Fig. 1, which consists of three modules, i.e. local feature learning module, causal representation learning module, subtasks prediction module. Generally, the input is the sequence *X* of each ARG sample, and the final output includes ${\hat{Y}}_{a}$, ${\hat{Y}}_{m}$, and ${\hat{Y}}_{t}$ which denotes the predicted probability of antibiotic class, resistance mechanism, and transferability, respectively. The detailed description of each module is presented in following subsections.

### 2.1 Local representation learning

In this module, a multi-layer Convolutional Neural Network (CNN) is used to capture the local feature representation of the input sample. This module consists of six convolutional layers, four max pooling layers (Graham 2014), two fully connected layers, and a dropout layer. Initially, the sequence *X* is converted into a one-hot matrix which is denoted as $X^{\prime }$. Subsequently, through the local representation learning module, the input sequence is transformed into an intermediate feature representation *H*.

More specifically, in our implementation of the local representation learning module, the number of convolution kernels in the first three convolutional layers is 32, 64, and 128, respectively, and the size of the convolution kernels is 40×4, 30×4, and 30×4, respectively. The number of convolution kernels in the last three convolutional layers is 256, and the size of the convolution kernels is 20×3. To enhance generalization and prevent overfitting, we incorporate a dropout layer with a dropout rate of 0.5.

### 2.2 Causal representation learning

As the analysis in previous section, relying solely on the representation *H* (i.e. the input sequence) of ARG is insufficient for predicting all the features of a sample. As inspired by the causal representation learning method for out-of-distribution recommendation (Wang *et al.* 2022), we introduce a hidden random variable *G* to denote the unobserved information to determine the properties of ARGs. A causal view of latent representation generation is utilized to complete the causal representation learning. More specifically, a Gaussian Mixture Model (GMM) (Reynolds 2009) is used to generate the unobserved information *G* for each sample, in which each component (a Gaussian distribution) denotes each type of unobserved information to determine the properties of ARGs. In this subsection, we will elaborate on this module from two aspects: (i) we introduce the process of the latent representation learning from a causal view; (ii) we present the approach to derive the posterior of GMM.

#### 2.2.1 Causal view of latent representation learning

**Figure 2.**
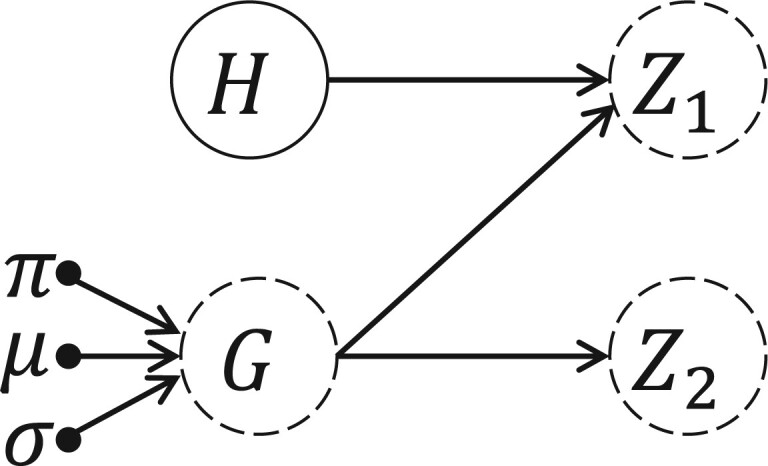
Causal graph of the Causal Representation Generation. Note that the solid circle denotes the observed variable, while dashed circles denote unobserved variables, and black solid dots represent parameters that need to be learned for GMM.

In this study, the process of causal representation generation is illustrated by the causal graph presented in Fig. 2. As shown in Fig. 2, the representation *H* obtained by Section 2.2 is viewed as the observed variable, which denotes the information contained in the input protein sequence. The random vector *G* denotes the unobserved information which is helpful to predict the properties of ARGs, and *G* is generated by a GMM with parameters (π,μ,σ). Specifically, $\pi \in R^{K}$ denotes the mixture coefficients of *K* components of GMM, while $\mu \in R^{d\times K}$ and $\sigma \in R^{d\times K}$ denote respectively the mean and diagonal covariance of *K* components of GMM, where *d* is the dimension of the random vector *G*.

Given each sample for predicting properties of ARGs, we assume that an effective latent representation consists of two parts: (i) $Z_{1}$ represents hidden features that are determined by both observed information (i.e. *H*) and unobserved information (i.e. *G*), such as gene expression levels and protein structures derived from gene expression; (ii) $Z_{2}$ represents hidden features that are determined only by unobserved information (i.e. *G*), such as the regulatory modes of gene expression. Consequently, the process of generating latent representation for each input sample (denoted by the hidden representation *H*) is summarized as follows.

1. Pick the index of the component from a Multinomial distribution with parameter π. Without loss of generality, assume *k* is picked;
2. Generate the unobserved variable *G* from the *k*th component of GMM, formally $G\leftarrow N(\mu_{k},\sigma_{k}\cdot I)$ where *N* denotes a Gaussian distribution;
3. Generate $Z_{1}$ from a function of *H* and *G*, formally $Z_{1}=f_{Z_{1}}(H,G)$;
4. Generate $Z_{2}$ from a function of *G*, formally $Z_{2}=f_{Z_{2}}(G)$.

Note that $f_{Z_{1}}(\cdot ,\cdot )$ and $f_{Z_{2}}(\cdot )$ are both implemented as neural network, parameters of which are learned in the training process of the whole framework. It is clear that the concatenation of $Z_{1}$ and $Z_{2}$ is the final representation for the prediction module, which is denoted by *Z*.

#### 2.2.2 Posterior of Gaussian mixture model

For the causal representation learning module, we need to learn the parameters of posterior of GMM based on the input training data. With the learned posterior of GMM, in the inference or testing phase, the unobserved variable *G* can be derived for the testing sample and the predicted properties of ARGs can be obtained accordingly.

In this study, we use neural networks to learn the parameters of GMM. Specifically, $f_{\phi }(\cdot ,\cdot )$ derives the mixing weights of GMM for each input sample. In other words, given the training sample (*X*, *Y*), the posterior distribution of the mixing weights of GMM is obtained by $f_{\phi }(X,Y)$, from which the component generating *G* can be picked from the corresponding Multinomial distribution. It is worth noting that during testing phase, in which only the sequence information *X* is available for each sample. In our implementation, therefore, we use the value of ‘-1’ (i.e. holding no meaning in our study) as the dummy *Y* to derive the corresponding variable *G* for each testing sample, the experimental results demonstrate that this process yields good enough performance.

With the similar treatment, the parameters of each Gaussian component in GMM are also approximated by a neural network. Formally, for the *k*th (k∈{1,…,K}) component, if it generates the unobserved variable for the given sample, the parameters of its posterior distribution are obtained by $f_{\theta_{k}}(\cdot ,\cdot )$. More specifically, given the training sample (X, Y), $f_{\theta_{k}}(X,Y)=(f_{\mu_{k}}(X,Y),f_{\sigma_{k}}(X,Y))\in R^{2d}$ derives the mean and diagonal covariance of the *k*th component Gaussian generating the sample (*X*, *Y*), in which the first *d* components $f_{\mu_{k}}(X,Y)$ denotes the mean vector and the last *d* components $f_{\sigma_{k}}(X,Y)$ denotes the diagonal covariance. Consequently, for each sample, the posteriors of *K* Gaussian mixtures are obtained by *K* neural networks, which are denoted by $f_{\theta_{1}}(\cdot ,\cdot ),f_{\theta_{k}}(\cdot ,\cdot ),\ldots ,f_{\theta_{K}}(\cdot ,\cdot )$. Given the training set of *N* samples $\left\{(X_{1},Y_{1}),\ldots ,(X_{n},Y_{n}),\ldots ,(X_{N},Y_{N})\right\}$, the mean of the posterior of the *k*th Gaussian component is computed as follows.

(1) $$\begin{array}{c} {\bar{\mu }}_{k}=\frac{\sum_{i=1}^{N}f_{\phi }^{k}(X_{i},Y_{i})\cdot f_{\mu_{k}}(X_{i},Y_{i})}{\sum_{i=1}^{N}f_{\phi }^{k}(X_{i},Y_{i})} \end{array}$$

where $f_{\phi }^{k}(X_{i},Y_{i})$ denotes the *k*th component of $f_{\phi }(X_{i},Y_{i})$, i.e. the mixing weight of the *k*th Gaussian for the training sample $\left(X_{i},Y_{i}\right)$.

In order to optimize the GMM, our objective consists of two parts. For the one part, we want to minimize the difference between posterior mean of each sample (e.g. $f_{\mu_{k}}(X_{i},Y_{i})$) and the average mean of the corresponding Gaussian component (e.g. $\mu_{k}$). It is reasonable that the more compact of each subgroup of unobserved variant *G*, the better of the modeling of unobserved information determining the properties of ARGs. It means that for samples with similar unobserved variant *G*, the properties of ARGs are partly determined by similar unobserved biological patterns. For the other part, we want to maximize the distance between the mean of posterior of different Gaussian components. The consideration is that different Gaussian components need to be clearly separated, which will enhance the model’s ability to capture distinctive unobserved biological patterns to induce bacteria antibiotic resistance.

According to the analysis mentioned above, the loss function for training GMM is defined as follows.

(2) $$\begin{array}{c} \begin{array}{c} \mathrm{Los}s_{\mathrm{GMM}}=\sum_{k=1}^{K}\frac{1}{N}\sum_{i=1}^{N}(f_{\mu_{k}}(X_{i},Y_{i})-{\bar{\mu }}_{k}){\left(f_{\mu_{k}}(X_{i},Y_{i})-{\bar{\mu }}_{k}\right)}^{T}\\ -\sum_{i=1}^{K}\sum_{j=i\;+\;1}^{K}({\bar{\mu }}_{i}-{\bar{\mu }}_{j}){\left({\bar{\mu }}_{i}-{\bar{\mu }}_{j}\right)}^{T} \end{array} \end{array}$$

It is clear that the first part in the righthand of Equation (2) is for the first part of the objective, while the second part in the righthand of Equation (2) is for the second part of the objective. It is worth noting that the GMM is trained in an iterative manner, and is trained with other parameters of the whole framework simultaneously. The details of training the whole framework will be presented in Section 2.4.

### 2.3 Subtask prediction

In this section, we use the hidden representation *Z* obtained from causal representation learning as the input for predicting the three properties of ARG. In order to integrate the causal relationships among different properties, we first obtain the causal graph of three prediction subtasks through conducting a causal relationship mining tool on the training set. Then, three prediction subtasks are performed by introducing the obtained causal relationships.

#### 2.3.1 Causal graph construction

Given the training data, a causal graph on three properties of ARGs is constructed by applying CausalNex (https://causalnex.readthedocs.io/en/latest/index.html), in which the DAGs with NO TEARS (Zheng *et al.* 2018) algorithm is used to transform the learning of the causal structure into learning a directed acyclic graph (i.e. Bayesian network) based on the dataset. The input of CausalNex is a matrix consisting of observed dataset, and the output is a DAG representing the causal relationships among properties of training data.

In our implementation, the input data is a matrix $Y\in R^{N\times 3}$, where *N* represents the number of samples in the training dataset, and three represents the three properties of ARGs. In matrix *Y*, each row represents a sample, and each column represents one of the three properties (i.e. antibiotic class, resistance mechanism and transferability). The output is the weight matrix of causal graph, which is denoted by $W\in R^{3\times 3}$, representing the directed relationships among the three properties of ARGs.

Initially, the weight matrix *W* is randomly initialized. Then, the gradient descent algorithm is used to minimize the designed loss function

(3) $$\begin{array}{c} \frac{1}{2n}||X-XW|{|}_{F}^{2}\;+\;\lambda ||W|{|}_{1}\;+\;\frac{\rho }{2}|h(W){|}^{2}\;+\;\eta h(W) \end{array}$$

in which there are three parts:

- Fit error: $\frac{1}{2n}||X-XW|{|}_{F}^{2}$ represents the difference between the predicted value of the model and the actual observed value.
- Structure error: $\lambda ||W|{|}_{1}$ is the $L_{1}$ regularization norm which is utilized to prevent overfitting of the model.
- Constraint: $\frac{\rho }{2}|h(W){|}^{2}\;+\;\eta h(W)$ serves as the constraint condition for learning DAG.

In addition, λ, ρ and η are hyper-parameters when training the method of causal graph construction. Note that the weight matrix *W* is optimized by using the gradient descent algorithm in an iterative manner.

After obtaining the optimized weight matrix *W*, the adjacency matrix $A\in R^{3\times 3}$ of causal graph can be constructed through the mapping operation shown in Eq.4. For more detailed derivation and proof of the algorithm, please refer to literature (Zheng *et al.* 2018). In addition, the setting of hyperparameters can also be found in the referenced work.

(4) $$\begin{array}{c} W_{ij}\neq 0\Leftrightarrow A_{ij}=1,W_{ij}=0\Leftrightarrow A_{ij}=0. \end{array}$$

In this study, after using CausalNex on the training samples, the learned causal graph is presented in the “Subtasks Prediction” module of Fig. 1. From Fig. 1, we can find that the antibiotic class is affected by both transferability and resistance mechanism, and the resistance mechanism is only affected by transferability. The causal relationships of three properties will be used for three predicting subtasks.

It is worth noting that the process of constructing causal graph is completed before training the whole framework, which can guarantee the efficiency of the optimizing the proposed method for predicting properties of ARGs.

#### 2.3.2 Prediction with causal graph

For each property of ARGs, we use multi-layer perceptrons (MLPs) to derive the predicted probability for given samples. In this study, after introducing causal associations among properties of ARGs, we observe that transferability can influence both resistance mechanism and antibiotic class, while the resistance mechanism can only affect the antibiotic class. Therefore, we will conduct predicting subtasks in the order of transferability, resistance mechanism, and antibiotic class. We treat each causal impression as an intervention (represented by do(·)). Therefore, predicting the resistance mechanism attribute can be seen as solving $P(M|Z,do(T={\hat{Y}}_{t}))$ while predicting the antibiotic class can be seen as solving $P(A|Z,do(T={\hat{Y}}_{t}),do(M={\hat{Y}}_{m}))$.

More specifically, given each sample, the predicted transferability $\hat{Y_{t}}$ is first derived by a multi-layer perceptron as shown in Equation (5). Then, the concatenation of $\hat{Y_{t}}$ and the hidden representation *Z* is input to a multi-layer perceptron $MLP_{M}(\cdot )$ for predicting resistance mechanism [as shown in Equation (6)], deriving the predicted resistance mechanism ${\hat{Y}}_{m}$. Finally, $\hat{Y_{t}}$, ${\hat{Y}}_{m}$, and *Z* are concatenated together and input to a multi-layer perceptron $MLP_{A}(\cdot )$ to predict the antibiotic class of the ARGs [as shown in Equation (7)].

(5) $$\begin{array}{c} {\hat{Y}}_{t}=MLP_{T}(Z) \end{array}$$

(6) $$\begin{array}{c} {\hat{Y}}_{m}=MLP_{M}([Z,{\hat{Y}}_{t}]) \end{array}$$

(7) $$\begin{array}{c} {\hat{Y}}_{a}=MLP_{A}([Z,{\hat{Y}}_{t},{\hat{Y}}_{m}]) \end{array}$$

### 2.4 Model training and testing

To further clarify the proposed approach, in this section, we provide a concise introduction to the training and testing phases of the overall framework. In the training phase, given the mini-batch of *N* samples $\left\{(X_{1},Y_{1}),\ldots ,(X_{n},Y_{n}),\ldots ,(X_{N},Y_{N})\right\}$ where $X_{n}$ is the sequence information of *n*th sample and $Y_{n}=(Y_{a},Y_{m},Y_{t})$ is the label of three properties.

For each sample $X_{n}$, the predicted labels are derived by Equations (5–7). The cross-entropy loss functions are defined respectively for these subtasks, which are presented as follows.

(8) $$\begin{array}{c} L_{t}=-\sum_{i=1}^{N}Y_{i,t}\;\text{log}\;{\hat{Y}}_{i,t} \end{array}$$

(9) $$\begin{array}{c} L_{m}=-\sum_{i=1}^{N}Y_{i,m}\;\text{log}\;{\hat{Y}}_{i,m} \end{array}$$

(10) $$\begin{array}{c} L_{a}=-\sum_{i=1}^{N}Y_{i,a}\;\text{log}\;{\hat{Y}}_{i,a} \end{array}$$

To optimize the proposed framework, the loss of three subtasks and loss of training GMM are combined as the final loss function which is a weighted sum of four loss functions. Formally, the final loss function is defined as follows.

(11) $$\begin{array}{c} \mathrm{Loss}=\alpha \cdot L_{a}+\beta \cdot L_{m}+\gamma \cdot L_{t}+\delta \cdot \mathrm{Los}s_{\mathrm{GMM}} \end{array}$$

where α, β, γ, and δ are hyper-parameters of the proposed method, which are utilized to adjust the weights of each loss for optimizing the model. In our implementation, by a grid search approach, the values of α, β, γ, and δ are set as 1, 0.2, 0.2, and 0.2, respectively.

## 3 Experiments

### 3.1 Dataset

In experiments, we use HMD-ARG-DB established by (Li *et al.* 2021) to evaluate the proposed method. The HMD-ARG-DB was collected by integrating ARG sequences from seven published antibiotic databases and removing duplicate sequences within them. After the preprocessing, a total of 17 282 high-quality ARG sequences are obtained, each of which are annotated by three properties: antibiotic class, resistance mechanism, and transferability. More specifically, there are 15 categories for antibiotic classes, and 6 categories for resistance mechanisms. Transferability is classified into two types: “transferable” and “intrinsic.”

### 3.2 Experimental setup

In order to demonstrate the superior performance of Causal-ARG, we use the following three prevailing baseline methods for comparative experiments: BestHit (Alcock *et al.* 2020), DeepARG (Arango-Argoty *et al.* 2018), and HMD-ARG (Li *et al.* 2021). Due to the limit of space, the detailed descriptions of baselines are provided in Supplementary Material. Note that parameters of each baseline are adjusted accordingly, and the parameter configuration that yields the optimal results is used for performance evaluation.

To assess performance, three commonly utilized evaluation metrics are applied in our experiments: precision (P), recall (R), and F1 score (F1). Considering the uneven distribution of categories for each property, the macro versions of P, R, and F1 are used. More specifically, the evaluation metrics (P, R, or F1) are computed for each category, and the average value across all categories is obtained as the final evaluation score for comparison.

In experiments, we conduct 5-fold cross-validation for model training. For each fold, the training set accounts for 80% of the data, while the validation set accounts for 20%. To avoid data bias, we repeat the each experiment ten times with different random initializations, and the average and standard deviation for each metric are computed for final performance comparison. In addition, to make a fair comparison, the setups for training, and testing division are completely the same across different methods.

### 3.3 Overall performance

In experiments, we compare the Causal-ARG with selected baselines on three subtasks of ARGs properties prediction, i.e. antibiotic class, resistance mechanism, and transferability. The corresponding results are presented in Tables 1–3, respectively. Note that in these Tables, the optimal outcome is highlighted in bold font.

**Table 1.** Performance comparison on ARG antibiotic class prediction.

Methods	*P*	*R*	F1
BestHit	0.983±0.005	0.452±0.003	0.585±0.004
DeepARG	0.914±0.005	0.757±0.003	0.814±0.003
HMD-ARG	0.950±0.005	0.847±0.005	0.893±0.005
Causal-ARG	0.902±0.005	0.896±0.005	0.899±0.005

**Table 2.** Performance comparison on ARG resistance mechanism prediction.

Methods	*P*	*R*	F1
BestHit	0.832±0.010	0.476±0.005	0.566±0.005
HMD-ARG	0.867±0.010	0.768±0.010	0.795±0.010
Causal-ARG	0.947±0.010	0.85±0.010	0.882±0.010

**Table 3.** Performance comparison on ARG transferability prediction.

Methods	*P*	*R*	F1
HMD-ARG	0.964±0.005	0.890±0.003	0.926±0.003
Causal-ARG	0.964±0.005	0.964±0.003	0.964±0.003

Generally speaking, from results in Tables 1–3, we can observe that Causal-ARG achieves the best overall performance among the existing methods. Specifically, for the subtask of antibiotic class prediction, although BestHit achieves the best precision, the recall of BestHit is extremely low (even below 0.5), leading to the worst F1 score among all methods. The main reason is that BestHit highly relies on existing databases, thus exhibiting weak generalization ability when identifying new ARGs, resulting in high false-negative rate and low recall. Similarly, DeepARG also relies on the alignment between samples and existing databases, leading to the low recall. However, owing to the automatic feature learning capability of deep learning, its performance still outperforms BestHit clearly. Due to the powerful feature extraction ability of CNNs, HMD-ARG performs better than BestHit and DeepARG on antibiotic class prediction. Although the precision of Causal-ARG is not the best, its recall and F1 score are the best among all methods. Especially for recall, Causal-ARG outperforms the current best method HMD-ARG by over 5%.

As the best baseline model, HMD-ARG performs slightly worse than Causal-ARG on antibiotic class prediction. However, HMD-ARG is a multi-task learning model implemented via hard parameter sharing among three prediction subtasks, leading to the issue of “seesaw phenomena” among the performance on three subtasks. In other words, HMD-ARG has superior predictive performance on antibiotics class, while its performance on the other two properties is relatively poor. From Tables 2 and 3, we can find that the performance of Causal-ARG is clearly better than HMD-ARG, without exhibiting the problem of “seesaw phenomena.” Note that DeepARG is not shown in Table 2, since it lacks the ability to predict the resistance mechanism of ARGs. Furthermore, among three baselines, only HMD-ARG has the ability to predict ARG transferability, so that Table 3 only shows the comparison results between HMD-ARG and Causal-ARG.

The comparative outcomes of overall performance, as illustrated in Tables 1–3 indicate that three properties characterize ARGs from distinct perspectives and the association among the three tasks of property prediction is relatively complex. Therefore, the performance among three subtasks cannot be ensured to obtain the optimal result through the implementation of representation learning with hard parameter sharing. In Causal-ARG, we attempt to capture the potential biological patterns of ARGs through causal representation learning module. Moreover, a causal graph composed of causal relationships among different properties is constructed to facilitate the prediction of subtasks, deriving best performance on all of three property prediction subtasks. The effectiveness of each components in the proposed method will be analyzed in depth in the following Section 3.4.

### 3.4 Ablation study

In this section, we conduct an ablation study to explore the contributions of main modules in Causal-ARG for performance improvement. More specifically, we evaluate the effectiveness of both causal representation learning and subtasks prediction with causal graph, and the results are shown in Table 4.

**Table 4.** The results of ablation study.

Method	Antibiotic class			Resistance mechanism			Transferability		
	*P*	*R*	F1	*P*	*R*	F1	*P*	*R*	F1
Causal-ARG	**0.902**	**0.896**	**0.899**	**0.947**	**0.850**	**0.882**	**0.964**	**0.964**	**0.964**
Causal-ARG w/o GMM	0.885	0.876	0.880	0.855	0.782	0.807	0.959	0.959	0.959
Causal-ARG w/o causal	0.883	0.884	0.882	0.906	0.849	0.874	0.960	0.960	0.960

The optimal outcome is highlighted in bold font.

#### 3.4.1 Contribution of causal representation learning

In Table 4, “Causal-ARG w/o GMM” represents the variant which removes the GMM in causal representation learning, i.e. the hidden representation *Z* is trained only via *H*. After removing the latent causal representation *G*, the performance of all subtasks has decreased clearly. In particular, the performance of the model in annotating resistance mechanism has significantly decreased, with all evaluation metrics falling by >5%. The possible reason is that learning hidden variable *G* through GMM can further capture the biological patterns which are not contained in sequence information, and these mechanisms contribute significantly to annotating resistance mechanisms of ARGs. This observation demonstrates that the causal representation learning module is beneficial for deriving high-quality representations of ARGs, which can help to improve the recognition accuracy of ARGs’ properties.

#### 3.4.2 Contribution of subtasks prediction with causal graph

In Table 4, “Causal-ARG w/o causal” represents the variant which removes the causal graph among three subtasks. In this variant, the hidden representation *Z* is input into three different fully connected network to predict three properties while ignoring the causality among them. From the results in Table 4, we can find that the performance of three subtasks is lower than our original method. More specifically, we can find that the prediction performance on resistance mechanism and antibiotic class decreases significantly, while the performance on predicting transferability is relatively stable. It may because that transferability affects both resistance mechanism and antibiotic class. Once the causal relationship among the three subtasks is removed, subtasks of predicting resistance mechanism and antibiotic class lose the supportive information from transferability, resulting in a decrease in prediction performance. This observation demonstrates that the causal graph on three subtasks is helpful for improving the performance of annotating properties of ARGs, especially for resistance mechanism and antibiotic class.

### 3.5 Influence of the number of Gaussian distributions in GMM

**Figure 3.**
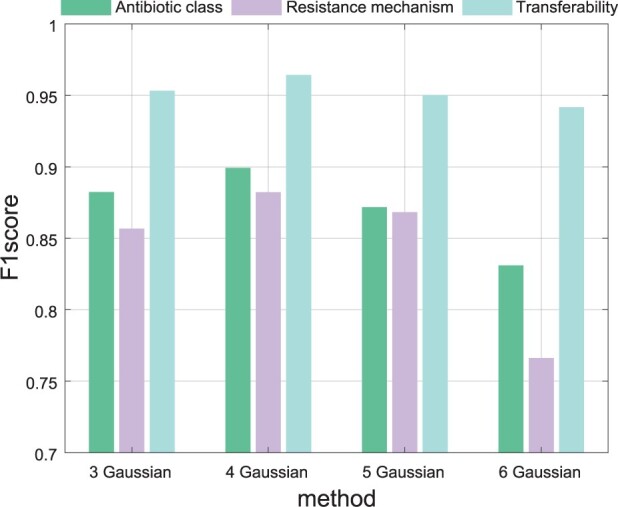
The results of models with different size of GMM.

To investigate the impact of GMM with different sizes on model performance, we test our model with varying numbers of Gaussian distributions in the GMM, from three to six, and the results are shown in Fig. 3.

From the Fig. 3, we can find that our model with four components in GMM achieves the best performance. More specifically, we have the following observations.

- When the number of Gaussian distributions in GMM is too small, the hidden variable *G* may struggle to capture the complexity and diversity of unobserved biological patterns inducing antibiotic resistance. Consequently, the learned representation (i.e. *Z*) might fail to describe complete semantics for determining properties of ARGs, resulting in a decline in model performance.
- On the contrary, GMM with too many Gaussian distributions may result in the issue of overfitting to the training data. Because each Gaussian distribution in GMM attempts to describe a locally compact region of hidden space, and excessive Gaussian components might capture meaningless features in the training data, leading to an undesirable performance. In addition, GMM with too many Gaussian distributions will make it more challenging for the model to converge during the optimization process.

### 3.6 Case study

#### 3.6.1 Visualization of unobserved variable *G*

**Figure 4.**
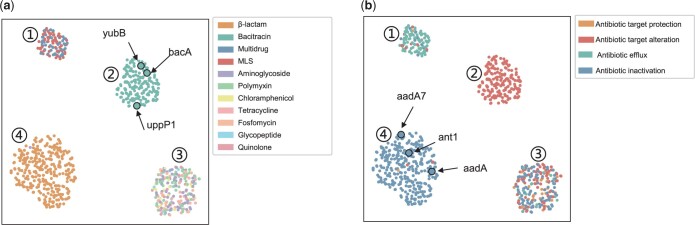
Visualization of unobserved variable *G*: display according to (a) antibiotic class and (b) resistance mechanism.

To further investigate the information captured by the unobserved variable *G*, we apply the t-SNE (Wattenberg *et al.* 2016) as dimensionality reduction algorithm, and plot the values of *G* for testing samples visually in Fig. 4a and b. In the visualization process, the samples are colored according to labels of different properties. As shown in Fig. 4a, the samples are colored differently based on their antibiotic classes. From Fig. 4a, we observe that the samples are distinctly divided into four clusters, demonstrating that four hidden biological patterns are identified by GMM. Furthermore, we can find that ARGs whose antibiotic classes are β-lactam and bacitracin are accurately assigned to two distinct clusters (i.e. cluster ID 4 and cluster ID 2, respectively), indicating that certain common biological patterns might determine the property of antibiotic class for ARGs with β-lactam and bacitracin. The remaining two clusters consists of ARGs with diverse antibiotic classes. The possible reasons lie in two aspects: (i) even though some ARGs belong to different categories of antibiotic classes, they might share common biological patterns which determine the bacteria antibiotic resistance; (ii) for some categories of antibiotic classes, the number of samples is too limited to derive meaningful hidden biological patterns (i.e. the variable *G*) for samples, resulting in a cluster containing multiple types of antibiotic classes. Note that we remove the categories of antibiotic classes with <30 samples in test set (i.e. “Thrimethoprim,” sulfonamide,’ “rifampin,” and “others”).

In addition, we also visualize the unobserved variable *G* according to different resistance mechanisms, as shown in Fig. 4b. From Fig. 4b, we can find that ARGs annotated with Antibiotic target alteration, Antibiotic efflux and Antibiotic inactivation are clearly grouped into three clusters (i.e. cluster ID 2, cluster ID 1, and cluster ID 4, respectively), which indicates that the unobserved variable *G* is capable of capturing the difference among ARGs with resistance mechanisms. Furthermore, we observe that the cluster associated with the Antibiotic efflux (i.e. cluster ID 1) contains multiple antibiotic classes in Fig. 4a. This phenomenon demonstrates that although the antibiotic classes of ARGs may not be the same, they share a common resistance mechanism, confirming our hypothesis mentioned before. It is worth noting that we remove the categories of resistance mechanisms with <30 samples in test set (i.e. “antibiotic target replacement” and “others”).

Based on above observations, we can conclude that introducing unobserved variable *G* enables us to learn the potential biological patterns behind ARGs, which plays a significant role for accurately annotating properties of ARGs.

#### 3.6.2 Analysis of specific cases

To validate the reliability of the underlying biological patterns captured by unobserved variable *G*, we select samples from the test set and conduct searches in the UniProt database to investigate the specific biological patterns of antibiotic resistance.

More specifically, regarding the property of antibiotic class, we retrieve three ARGs which are resistant to Bacitracin, and the corresponding unobserved variables are marked with black outlined circles in Fig. 4a. The detailed information of these samples is presented in Table 5. All these three ARGs confer bacteria resistance to bacitracin by catalyzing the dephosphorylation of undecaprenyl diphosphate (Manat *et al.* 2014).

**Table 5.** Specific cases for antibiotic class.

ARGs	Antibiotic class	Underlining biological pattern
yubB	Bacitracin	Catalyzes the dephosphorylation of undecaprenyl diphosphate to confer resistance to bacitracin
uppP1	Bacitracin	
bacA	Bacitracin	

For the property of resistance mechanism, we take the same treatment, and select three ARGs with the resistance mechanism of “antibiotic inactivation.” The corresponding unobserved variables are marked with black outlined circles in Fig. 4b. According to the description in Table 6, we know that the unobserved variable *G* can capture the common reason to determine the resistance mechanism, i.e. catalyzing the reaction between ATP and spectinomycin (Stern *et al.* 2018) to generate substances which inactivate antibiotics.

**Table 6.** Specific cases for resistance mechanism.

ARGs	Resistance mechanism	Underlining biological pattern
aadA7	Antibiotic inactivation	Catalyze the reaction between ATP and spectinomycin to generate substances that can inactivate antibiotics
aadA	Antibiotic inactivation	
ant1	Antibiotic inactivation	

## 4 Conclusions

In this study, we have proposed a novel framework for annotating properties of ARGs, in which causality of ARG properties is utilized for representation learning. Specifically, a GMM is used to model the unobserved factors to determine the multiple properties of ARGs. Moreover, a causal graph between different properties of ARGs is constructed by a tool of causal relationship mining, deriving the improved performance on annotating three properties of ARGs. Results on a real-world dataset demonstrate the proposed method outperforms the state-of-the-art methods on annotate ARGs’ properties, and the ablation experiments validate the effectiveness of the main components in our model as well. In addition, the results of case study suggest that the unobserved variables in our model can capture the underlying biological patterns which affects the properties of ARGs.

## Supplementary Material

btae180_Supplementary_Data
