# Supplementary material for: Causal-ARG: a causality-guided framework for annotating properties of antibiotic resistance genes
Source: Bioinformatics. 2024 Apr 3;40(4):btae180. doi: 10.1093/bioinformatics/btae180 (PMC11026140; doi:10.1093/bioinformatics/btae180)
Supplement: btae180_Supplementary_Data [file btae180_supplementary_data.pdf]

Supplementary Material for "Causal-ARG: A Causality-guided Framework for Annotating Properties of Antibiotic Resistance Genes"

Baselines

To comprehensively evaluate the effectiveness of the proposed method, the following three representative methods are selected as baselines for performance comparison:

BestHit: This method is conducted by comparing the sample sequences with existing ARGs in CARD by applying the BLAST or DIAMOND, and the predicted properties are assigned to samples through applying a similarity cutoff. Note that BestHit can be used only for predicting antibiotic classes and resistance mechanisms of ARGs. For detailed usage of BestHit, please refer to the link: https://card.mcmaster.ca/analyze/blast.

DeepARG: This method is a deep learning-based model which is trained by taking the consistency distribution of homologies between sample sequences and all known ARGs as input features. Note that DeepARG can be used only for predicting antibiotic classes of ARGs. For detailed usage of the DeepARG, please refer to the link: https://github.com/gaarangoa/deeparg.

HMD-ARG: This method extracts features from raw sequences through an end-to-end deep CNN-based framework for predicting properties of ARGs. Note that HMD-ARG is a multi-task model, which can be used for predicting all of three properties of ARGs. For detailed usage of the HMD-ARG, please refer to the link: http://www.cbrc.kaust.edu.sa/HMDARG.
